# Optimising the antibiotic treatment of uncomplicated acute appendicitis: a protocol for a multicentre randomised clinical trial (APPAC II trial)

**DOI:** 10.1186/s12893-018-0451-y

**Published:** 2018-12-17

**Authors:** J. Haijanen, S. Sippola, J. Grönroos, T. Rautio, P. Nordström, T. Rantanen, M. Aarnio, I. Ilves, S. Hurme, H. Marttila, J. Virtanen, A. Mattila, H. Paajanen, P. Salminen, on behalf of the APPAC study group, J. Rintala, S. Meriläinen, J. Laukkarinen, H. Savolainen, T. Pinta, E-L Sävelä, T. Sippola, P. Böckerman

**Affiliations:** 1 Division of Digestive Surgery and Urology, Turku University Hospital, Kiinanmyllynkatu 4-8, 20520 Turku, Finland; 2 Department of Surgery, University of Turku, Turku, Finland; 3 Department of Surgery, Satakunta Central Hospital, Pori, Finland; 4 Department of Surgery, Oulu University Hospital, Oulu, Finland; 5 Division of Surgery, Gastroenterology and Oncology, Tampere University Hospital, Tampere, Finland; 6 Department of Surgery, Kuopio University Hospital, Kuopio, Finland; 7 Department of Surgery, Seinäjoki Central Hospital, Seinäjoki, Finland; 8 Department of Surgery, Jyväskylä Central Hospital, Jyväskylä, Finland; 9 Department of Surgery, Mikkeli Central Hospital, Mikkeli, Finland; 10 Department of Biostatistics, University of Turku, Turku, Finland; 11 Department of Hospital Hygiene and Infection Control, Turku University Hospital, Turku, Finland; 12 Department of Radiology, Turku University Hospital, University of Turku, Turku, Finland; 13 Institute of Clinical Medicine, University of Eastern Finland, Kuopio, Finland

**Keywords:** Appendicitis, Acute appendicitis, Uncomplicated appendicitis, Antibiotic therapy, Non-operative treatment, Low-dose CT

## Abstract

**Background:**

Based on epidemiological and clinical data acute appendicitis can present either as uncomplicated (70–80%) or complicated (20–30%) disease. Recent studies have shown that antibiotic therapy is both safe and cost-effective for a CT-scan confirmed uncomplicated acute appendicitis. However, based on the study protocols to ensure patient safety, these randomised studies used mainly broad-spectrum intravenous antibiotics requiring additional hospital resources and prolonged hospital stay. As we now know that antibiotic therapy for uncomplicated acute appendicitis is feasible and safe, further studies evaluating optimisation of the antibiotic treatment regarding both antibiotic spectrum and shorter hospital stay are needed to evaluate antibiotics as the first-line treatment for uncomplicated acute appendicitis.

**Methods:**

APPAC II trial is a multicentre, open-label, non-inferiority randomised controlled trial comparing per oral (p.o.) antibiotic monotherapy with intravenous (i.v.) antibiotic therapy followed by p.o. antibiotics in the treatment of CT-scan confirmed uncomplicated acute appendicitis. Adult patients with CT-scan diagnosed uncomplicated acute appendicitis will be enrolled in nine Finnish hospitals. The intended sample size is 552 patients.

Primary endpoint is the success of the randomised treatment, defined as resolution of acute appendicitis resulting in discharge from the hospital without the need for surgical intervention and no recurrent appendicitis during one-year follow-up. Secondary endpoints include post-intervention complications, late recurrence of acute appendicitis after one year, duration of hospital stay, pain, quality of life, sick leave and treatment costs. Primary endpoint will be evaluated in two stages: point estimates with 95% confidence interval (CI) will be calculated for both groups and proportion difference between groups with 95% CI will be calculated and evaluated based on 6 percentage point non-inferiority margin.

**Discussion:**

To our knowledge, APPAC II trial is the first randomised controlled trial comparing per oral antibiotic monotherapy with intravenous antibiotic therapy continued by per oral antibiotics in the treatment of uncomplicated acute appendicitis. The APPAC II trial aims to add clinical evidence on the debated role of antibiotics as the first-line treatment for a CT-confirmed uncomplicated acute appendicitis as well as to optimise the non-operative treatment for uncomplicated acute appendicitis.

**Trial registration:**

Clinicaltrials.gov, NCT03236961, retrospectively registered on the 2nd of August 2017.

## Background

Acute appendicitis is the most common cause of abdominal pain in emergency departments. The lifetime risk of acute appendicitis in males is 8.6 and 6.7% in females [1] with recent meta-analysis showing an increasing trend in the incidence of appendicitis in the newly industrialised countries [2]. Appendectomy has unquestionably been the standard treatment for acute appendicitis for over a century with more than 300.000 appendectomies performed annually in the United States [3]. Although appendectomy is generally well tolerated, it is a major surgical intervention and can be associated with postoperative morbidity [4–6]. Recently, an increasing amount of evidence has been reported showing that the majority of patients with uncomplicated acute appendicitis may be treated with antibiotics alone instead of surgery [7–11]. The APPAC trial reported that 73% of patients with uncomplicated acute appendicitis treated with antibiotics did not require appendectomy during a 1-year follow-up period, and those patients who required appendectomy did not experience major complications suggesting that CT-proven uncomplicated acute appendicitis is not a surgical emergency and that antibiotic treatment is a safe first-line treatment for uncomplicated acute appendicitis [10]. This view has since been endorsed in recent meta-analyses [7–9, 12]. The economic evaluation from the APPAC trial at one-year follow-up also showed substantial cost savings of 2245€ per patient favouring the antibiotic treatment over appendectomy [13].

The difficulty and variability of determining the treatment efficacy, success or failure, for two different treatment modalities presents a significant bias in the randomised studies and the reported treatment success of antibiotic therapy is naturally inferior to appendectomy when evaluated only by treatment efficacy. However, we need to look beyond this overly-confining assessment by treatment success only and acknowledge that the clinical dilemma is more complicated including multiple factors to be weighed by clinicians, patients, and payors, i.e. the question is more about choosing the optimal treatment for each patient.

Based on large epidemiological studies, we now know that complicated and uncomplicated appendicitis have followed different epidemiological trends suggesting different pathophysiology for the two forms of acute appendicitis [3]. The majority (approximately 70–80%) of acute appendicitis cases are of uncomplicated nature [14, 15] which may not require surgical intervention and might even experience spontaneous resolution without perforation [3, 16, 17]. Complicated acute appendicitis, defined as a finding of perforation, appendicolith, abscess or a suspicion of tumor [10] requires emergency appendectomy with the exception of cases with abscess as they are often initially managed conservatively.

Although suspicion of acute appendicitis is the most common reason for surgical emergency visit, its diagnosis still remains challenging. The diagnosis has previously been based on patient history, clinical surgical diagnosis and laboratory findings. Several scoring systems have been created to aid in the diagnosis of acute appendicitis [18–20], but the accuracy of clinical diagnosis without preoperative imaging is still suboptimal for combined patient groups of males and females [15]. Furthermore, neither the clinical findings nor laboratory markers are reliable enough to establish the clinically relevant differential diagnosis between uncomplicated and complicated acute appendicitis without CT imaging [21].

Promising results of antibiotic treatment of uncomplicated acute appendicitis underline this vital importance of preoperative differential diagnosis between uncomplicated and complicated forms of acute appendicitis. Of the imaging modalities generally available in the emergency rooms, CT has been shown superior to ultrasound in diagnosing acute appendicitis [22–24]. CT has been shown feasible also for the differentiation between uncomplicated and complicated appendicitis [21, 22, 25]. Novel scoring systems combining clinical and radiological parameters have also been created to aid in the differential diagnosis between uncomplicated and complicated appendicitis highlighting the vital clinical importance of accurate diagnosis of appendicitis severity [26–29]. The advantages of CT imaging are high accuracy, availability, reproducibility, ease of performance and interpretation, and that it is rarely affected by bowel gas, severe abdominal pain or extreme body habitus [30, 31]. The increased use of preoperative CT imaging is also cost efficient through increased diagnostic accuracy and the avoidance of unnecessary appendectomies [13, 32, 33]. The inevitable disadvantage of CT imaging is exposure to ionising radiation potentially increasing future cancer risk [34], and as the majority of patients with acute appendicitis are young adults, the imminent need to decrease radiation dose has been approached through developing low-dose CT protocols [35]. Despite recent meta-analysis [36] showing equal accuracy of low-dose and standard CT in diagnosing acute appendicitis, the low-dose CT protocols have not yet been thoroughly implemented in clinical practise. Our study group conducted a prospective inter-patient randomised observational study, the OPTICAP trial [37], in which the same patient with suspected acute appendicitis underwent protocol sequence randomised consecutive imaging with both an empirically optimised 120 kV standard and a 100 kV low-dose CT protocols with intravenous contrast media. The primary objective of the OPTICAP trial was first to find the most optimal low-dose CT protocol in the phantom model in in vitro-phase of the OPTICAP trial [38], and then to test the accuracy of this low-dose protocol in diagnosing and subcategorising acute appendicitis in the clinical phase of the study. A body mass index (BMI) of 30 kg/m^2^ was set as an upper limit for low-dose CT imaging to avoid additional image noise and simultaneous unfavorable dose increase especially at the lower voltages due to increased tissue attenuation in larger patients. Based on the OPTICAP results, the most optimal low-dose protocol was implemented to be used in all APPAC II trial hospitals for the enrolment of APPAC II trial.

All the conducted RCTs [10, 11, 39, 40] comparing appendectomy with antibiotic treatment differ considerably in terms of the antibiotic treatment (regime, duration and administrative routes), definition of primary outcome and the diagnostic criteria for uncomplicated acute appendicitis with only two studies [10, 11] using CT-confirmed uncomplicated acute appendicitis as an inclusion criterion. In clinical practice, the differential diagnosis between uncomplicated and complicated acute appendicitis is crucial, and computed tomography (CT) imaging is essential in distinguishing between the two forms [21]. In order to enable comparison of imaging results, histopathology and treatment options, standardised CT criteria for appendicitis severity need to be established as differences in definitions for appendicitis severity at CT prevent true comparison of different studies. As noted by Vons et al. [11], a finding of an appendicolith in preoperative imaging is associated with higher rates of antibiotic treatment failure and if they had excluded the patients with appendicoliths in their study, no significant difference in postintervention peritonitis would have been found between the antibiotic and appendectomy groups. Another limitation of the prior antibiotic trials for treating appendicitis was the selection of the antibiotic. Vons et al. [11] used amoxicillin-clavulanic acid, although this combination is associated with considerable Escheria coli nonsusceptibility, whereas in the APPAC trial [10] i.v. ertapenem was used to ensure a broad spectrum pathogen coverage. However, using broad spectrum intravenously administered antibiotics such as ertapenem increases the risk of developing antibiotic resistance as well as results in patient hospitalization adding the need of hospital resources. As the antibiotic treatment for uncomplicated acute appendicitis has now been proven safe, the emphasis of future research should be in optimising the antibiotic treatment in terms of both antibiotic spectrum and shorter hospital stay in order to evaluate the non-operative treatment as the first-line treatment for uncomplicated acute appendicitis.

To our knowledge, there are so far no RCTs comparing p.o. antibiotic monotherapy with i.v. antibiotic therapy followed by p.o. antibiotics in the treatment of uncomplicated acute appendicitis.

The APPAC II trial aims to further clarify the debated role of antibiotics as the first-line treatment for a CT-confirmed uncomplicated acute appendicitis as well as to optimise the non-operative treatment for uncomplicated acute appendicitis. In the APPAC II trial, we aim to evaluate p.o. antibiotic monotherapy compared with i.v. + p.o. antibiotic therapy in terms of treatment efficacy, post-intervention complications, length of hospital stay and treatment costs.

## Methods

### Study design

**Fig. 1:**
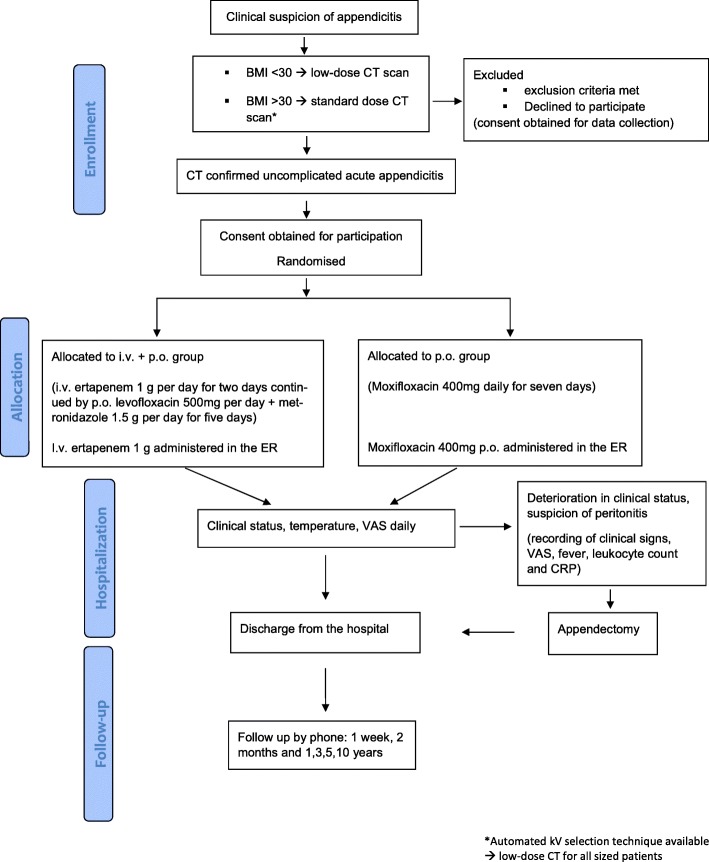
APPAC II flow diagram

The APPAC II trial is a randomised open-label, non-inferiority multicentre trial comparing per oral antibiotic monotherapy with intravenous antibiotic therapy followed by per oral antibiotics in the treatment of CT confirmed uncomplicated acute appendicitis. The aim of the study is to optimise the antibiotic treatment for uncomplicated acute appendicitis as well as to evaluate the antibiotic treatment as the first-line treatment for uncomplicated acute appendicitis in clinical practise. The protocol was drafted in accordance with the SPIRIT (Standard Protocol Items: recommendations for Interventional Trials) statement [41]. The trial has been approved by the Ethics Committee of the Hospital District of Southwest Finland and the Finnish Medicines Agency (Fimea), and has been registered at clinicaltrials.gov (NCT03236961). All patients participating in the study will give written consent. The study flow is illustrated in Fig. 1.

### Patient selection and diagnostic procedures

Eligible for inclusion are adult patients 18–60 years old of both sexes admitted to the emergency department of one of the participating hospitals with suspected acute appendicitis in whom a CT-confirmed uncomplicated acute appendicitis is diagnosed.

All adult patients with a clinical suspicion of acute appendicitis will be studied carefully by attending surgeons at the emergency departments of the participating hospitals. Clinical history, physical investigation, and laboratory tests (blood haemoglobin, white blood cell count, C-reactive protein (CRP), creatinine, urine analysis and in female patients also human chorionic gonadotropin) are evaluated. Prior to pain medication administration, pain scores using visual analogue scale (VAS) will be recorded.

If clinical history and physical examination suggest acute appendicitis, the patient will undergo CT imaging with either an optimised 100 kV [38] low-dose (BMI < 30 kg/m^2^) or standard 120 kV contrast enhanced CT (BMI >  30 kg/m^2^). Alternatively, a corresponding low-dose protocol with automated tube voltage (kV) selection was used in all sized patients, if the technique was available in the study hospitals.

#### Inclusion criteria

The inclusion criteria are: 1) Age 18–60 years, 2) Diagnosis of uncomplicated acute appendicitis confirmed by CT scan defined by the following criteria: appendiceal diameter exceeding 6 mm with thickened and enhancing wall and periappendiceal edema and /or minor fluid collection, and the absence of the criteria of complicated appendicitis. The CT findings will be evaluated using a standardised CT scan report sheet (Table 1).

**Table 1:** Structured reporting template of abdominal CT: APPAC multicenter

I Descriptive part of the report: Technique and findings in the whole abdomen	
II Structured report of appendix: 1) Appendix Visualisation	
Report one of the following:	
Not visualised/ Partly or unclearly visualised/ Completely visualised	
2) Appendix transverse diameter (mm):	
3) Probability of appendicitis	
Report one of the following:	
Not likely/ Rather unlikely/ Rather likely/ Very likely	
4) Categorisation of the appendicitis	
Report either I or II, if any:	
I Uncomplicated appendicitis: transverse diameter > 6 mm with typical findings	
-wall thickening and enhancement	
-periappendiceal edema and/or minor amount of fluid	
II Complicated appendicitis: Appendicitis with at least one of the following:	
-Appendicolith: > 3 mm stone within appendix	
-Abscess: periappendiceal walled of collection with enhancing walls	
-Perforation: appendiceal wall enhancement defect and periappendiceal excess of fluid and/or infectious phlegmon and/or extraluminal air	
-Tumor: tumor-like prominence of appendix	
Other diagnosis: Report if any	
Diverticulitis/ Complicated ovarian cyst/ Pelvic inflammatory disease/ Colitis/ Ileitis /Intestinal obstruction or ileus/ Ureter stone/ Hydronephrosis/ Tumor/ Other diagnosis	

#### Exclusion criteria

The exclusion criteria are: 1) Age under 18 or over 60 years, 2) Pregnancy or lactation, 3) Allergy to contrast media or iodine, 4) Allergy or contraindication to antibiotic therapy, 5) Renal insufficiency or serum creatinine value exceeding the upper reference limit, 6) Type 2 diabetes mellitus and metformine medication, 7) Severe systemic illness (for example malignancy, medical condition requiring immunosuppressant medication), 8) Inability to co-operate and give informed consent, and 9) Complicated acute appendicitis in the CT scan. A radiological diagnosis of complicated acute appendicitis is defined as typical findings of appendicitis with at least one of the following: appendicolith, periappendiceal abscess, perforation, or suspicion of an appendiceal tumor (Table 1). Contraindications for the use of antibiotics include either allergy to the antibiotic regimen, auxiliary substance of the drug, or interaction with other medications of patient. In the case of quinolones, epilepsy and previously diagnosed tendinitis or tendon rupture related to quinolone treatment are contraindications. With moxifloxacin, additional contraindications are liver failure (also a general contraindication for study enrolment), heart condition (for example prolonged QT-time) and electrolyte imbalance. Other overall contraindications to antibiotic treatment in general including pregnancy or lactation and age under 18 years, do not apply as these patients will not be evaluated for study enrolment based on general exclusion criteria.

#### Excluded patients

If complicated acute appendicitis is diagnosed, patients will undergo emergency appendectomy as soon as possible according to standard care and antibiotic therapy will be initiated already at the emergency department, if evaluated necessary by the attending surgeon. Other diagnoses at the abdominal CT scan will be treated according to the standard care.

In order to both prevent any selection bias and to enable a thorough recording of the patient population with suspected acute appendicitis, all patients undergoing a CT for suspected acute appendicitis will be recorded in the study online database. All patients will be informed about data collection and they will sign an informed consent for this data collection. Clinical history, physical investigation, VAS pain score and laboratory tests will be recorded in the study online database.

### Randomisation

Patients are randomised with a 1:1 equal allocation ratio to p.o. or i.v. + p.o. antibiotic group. Randomisation will be made by a safety statistician of the trial by center using random permuted blocks. After written informed consent, randomisation of the patient will be opened using the online database by the surgeon on call in each participating hospital. The randomised treatment will be initiated in the emergency room.

### Sample size calculation

As the primary objective of the study is to demonstrate that p.o. antibiotics are non-inferior compared to a combination of i.v. and p.o. antibiotic therapy, a non-inferiority design is used and sample size calculations were based on non-inferiority test for binomial proportion. Sample size was calculated from an estimated success rate of 73% for i.v. + p.o. antibiotic group during the one year follow-up based on the results of our previous APPAC trial [10]. The hypothetical difference between the two groups ((i.v. + p.o.) – (p.o.)) was set to zero and non-inferiority margin was set to 6 percentage points. We estimated that a total of 469 patients would yield a power of 0.9 (1-β) to establish whether p.o. antibiotic therapy was non-inferior to i.v. + p.o. using a one-sided significance level (α) of 0.05. With an estimated dropout rate of 15% total of 552 patients, 276 patients per group will be enrolled in the study. Targeted minimum sample size per study hospital will be 20 patients.

### Study setting and feasibility

Eligible patients are recruited in nine participating hospitals across Finland; four university hospitals (Turku, Tampere, Oulu, and Kuopio) and five central hospitals (Jyväskylä, Mikkeli, Pori, Seinäjoki, and Rovaniemi). The study was initiated at the Turku and Kuopio University Hospitals in April 2017, with the study commencing at all the study centres by fall 2017. Patient recruitment is evaluated to be completed by August 2018.

### Interventions

The duration of the antibiotic treatment in both treatment groups will be seven days and the allocated treatment will be initiated in the emergency room. The minimum follow-up at the hospital will be 20–24 h depending on time of day at admission and patient status.

#### Per oral antibiotic group

Per oral antibiotic treatment consists of moxifloxacin 400 mg once daily for seven days, the first two doses will be given while patient is in the hospital.

#### Intravenous + per oral antibiotic group

Intravenous antibiotic followed by per oral antibiotic therapy consists of two days of i.v. ertapenem sodium 1 g a day followed by p.o. levofloxacin 500 mg a day and metronidazole 500 mg three times a day for five days. The minimum follow-up at the hospital enables the i.v. treatment for two days.

### Follow-up during the hospitalisation

During the hospitalisation the following parameters will be recorded every 24 h: VAS or changes in VAS, leukocyte count, C-reactive protein, temperature and clinical findings at patient reassessment. A surgeon will reassess the patient twice daily. Pain medication is prescribed according to standard hospital protocol. If the patient is suspected of not responding to the antibiotic therapy based on clinical deterioration signs combined with laboratory findings (signs of peritonitis, persisting fever, increasing level of pain, rising white blood cell count or CRP), the patient will be operated on based on the surgeon’s decision and the reasons for proceeding to appendectomy will be recorded in the database. For appendectomy, laparoscopic approach is recommended. The operative findings and the histopathology of the appendix will be recorded in the database.

After the initial hospitalisation recurrent acute appendicitis will be diagnosed on a clinical basis and patients with a suspected appendicitis recurrence will undergo a laparoscopic appendectomy. The operative findings and the histopathology of the appendix will be recorded in the database.

## Outcome parameters and statistical analysis

### Primary outcome

The primary endpoint of the APPAC II trial is defined as the success of the randomised treatment at one-year follow-up (treatment efficacy). Treatment success in both groups is defined as the resolution of acute appendicitis with antibiotic treatment resulting in discharge from the hospital without the need for surgical intervention and no recurrent appendicitis during a follow-up of one year.

### Secondary outcomes

Secondary endpoints include post-intervention complications (possible postoperative complications classified using the Clavien-Dindo classification [42]), late recurrence (after one year) of acute appendicitis after antibiotic treatment, duration of hospital stay, VAS scores, quality of life (QOL, using for example 5D or 15D validated QOL questionnaire), length of sick leave and treatment costs.

### Data collection

Data collection from all patients presenting with suspected acute appendicitis in the emergency room includes: tympanic temperature, nausea and its duration, pain in the right lower quadrant of the abdomen and its duration, pain migration, presence of tenderness in the right lower quadrant, and the VAS score. Additionally, CT scan results and laboratory test results will be recorded. Daily follow-up data of study patients with acute appendicitis during hospitalisation will include status findings, VAS score, tympanic temperature as well as white blood cell count and C-reactive protein laboratory results. At discharge, the length of hospital stay, VAS score, profession, length of sick leave and the prescribed analgesics are recorded.

If the patient undergoes appendectomy for either suspected non-responsiveness to antibiotic therapy during primary hospitalisation or for suspected recurrence after initial successful antibiotic treatment, operative details including operation duration, approach, and operative findings will be recorded. Possible wound infection and possible postoperative antibiotic therapy will be evaluated and recorded upon discharge.

After discharge, the follow-up for patients enrolled in the APPAC II trial will include a contact by telephone at one week, two months and at one, three, five and ten years. Data collected during the follow-up includes possible complications of the treatment given including also possible appendectomy, the possible recurrence of appendicitis and the timing of the recurrence with operative findings and histopathology of the removed appendix, VAS score at control time point, additional sick leave, quality of life, and abdominal symptoms evaluation. In case of patient reporting abdominal symptoms at the phone interview by the study group research nurse, the patient will be assessed by a surgeon and further clinical assessment including imaging and laboratory tests will be performed with a low threshold.

### Statistical hypothesis

The primary objective of the study is to demonstrate that p.o. antibiotics are adequate treatment for uncomplicated acute appendicitis. The primary outcome is success of treatment and it will be evaluated in two stages using following statistical hypotheses:

1. H_0_: p_1_ ≤ 65 and p_2_ ≤ 65

H_1_: p_1_ > 65 and p_2_ > 65

2) H_0_: p_1_ - p_2_ > 6

H_1_: p_1_ - p_2_ ≤ 6.

where p_1_ is success of treatment proportion of i.v. + p.o. group and p_2_ for p.o. group and p_1_ - p_2_ is difference between groups ((i.v. + p.o.) – po).

### Data analysis plan

Categorical variables of the study will be characterised by treatment using frequencies and percentages and for continuous variables means and standard deviations or medians with range and 25th and 75th percentiles will be used. The point estimate with 95% confidence interval (CI) for success of treatment will be calculated for both groups and if lower limit of 95% CI ≥ 65% then treatment is good enough. Non-inferiority of p.o. antibiotics vs. i.v. + p.o. antibiotics will be evaluated using a two-sided 90% CI of proportion difference between groups ((i.v. + p.o.) – p.o. antibiotics) and one-sided Wald test for non-inferiority with an α level of 0.05. Non-inferiority margin for difference is 6 percentage points. The secondary outcomes will be analysed using chi-squared test, independent samples t-test or Mann-Whitney U-test. The assumptions of tests will be checked for justification of the analyses. For the secondary outcomes two-sided *p*-values will be used. The study site differences will be evaluated in statistical models and if major differences are detected more complicated statistical models will be used in the analyses of primary and secondary outcomes. *P*-values less than 0.05 will be considered statistically significant. The analyses will be based on the intention-to-treat (ITT) principle (all randomised excluding possible erroneously randomised patients with CT diagnosis of complicated appendicitis). For the primary end-point, in cases of patients lost to follow-up, missing data will be gathered from hospital registries, if possible, but for secondary outcomes, the subjects with missing data will automatically be excluded from the analyses of the variables in concern. Sample size calculations were performed and statistical analyses will be performed using SAS System for Windows, Version 9.4 or later (SAS Institute Inc., Cary, NC).

### Interim analysis

When 250 patients are enrolled to the study and discharged from the hospital, or if the investigators think it is necessary, the point estimate of the success rate at discharge will be calculated by study statistician and evaluated in each group to ensure patient safety. If the proportion is below 70% in at least one of the groups, the study will be terminated. The whole study group will be informed of the group proportions and whether the study is allowed to continue or will be terminated. No statistical tests will be conducted in interim analysis and therefore no corrections to the *p*-values are needed in the final analyses of the study.

### Data collection and confidentiality

The researchers together with BCB Medical have created the online database, where all patients evaluated for acute appendicitis and study enrolment will be recorded after signed informed consent is obtained. All data are handled confidentially and the information in the datasets is non-identifiable. Data are gathered during emergency room visit, hospitalisation for acute appendicitis, clinical observations, and follow-up phone calls. The information recorded from the non-participating patients is used as register-based study data. The main investigators will be in charge of the common database with full access to the data, otherwise the access to the data is strictly limited. The researchers need full access to the data in order to be able to correct possible false data entries, to enter missing data and to be able to keep up with the number of enrolled patients. The online database will not be used for other purposes during the trial and all of the visits to the database will be recorded in the database log. In order to prevent selection bias we designed the study protocol to record data on all patients evaluated for eligibility.

### Withdrawal

Patients are informed of their right to withdraw from the study without explanation at any time. In case of patient withdrawal, they will be asked for permission to use their data.

### Dissemination plan

The results of this trial will be disseminated by publication in international peer-reviewed scientific journals and by presentations at international and/or domestic conferences. If the trial results warrant changes in the standard treatment of uncomplicated acute appendicitis, the widespread execution of the trial throughout Finland will assist in its implementation.

## Discussion

As non-operative treatment for uncomplicated acute appendicitis has been shown to be safe [7–12] and cost-effective [13], the time has come to evaluate optimisation of the antibiotic therapy in the treatment of uncomplicated acute appendicitis focusing on both required antibiotic spectrum and the antibiotic therapy associated hospital stay. The primary aim of this study is to optimise the antibiotic therapy for uncomplicated acute appendicitis with the hypothesis that p.o. antibiotics are non-inferior to a combination of i.v. and p.o. antibiotic therapy. Secondary aim is to evaluate antibiotic therapy as the first–line treatment in clinical practise for uncomplicated acute appendicitis in a large prospective patient cohort.

If our hypothesis is correct and this study can demonstrate the non-inferiority of p.o. antibiotics to combined i.v. and p.o. antibiotic therapy, the need for hospital resources would substantially decrease. The first pilot for outpatient treatment of uncomplicated acute appendicitis has already been conducted [43] and an outpatient approach or only short hospitalisation would presumably result in further cost savings and better utilisation of hospital resources related to antibiotic treatment of uncomplicated acute appendicitis.

### Choice of the primary outcome

The success of antibiotic treatment defined as resolution of acute appendicitis with antibiotic treatment resulting in discharge from the hospital without the need for surgical intervention and no recurrent appendicitis during a follow-up of one year was also used as the primary outcome in our previous APPAC trial [10]. Based on these results, the primary endpoint definition is clear, easy to measure, and comparable with our previous results, although we do acknowledge that this definition of the primary endpoint only takes into account the recurrences occurring during the first year. The long-term results of non-operative treatment for uncomplicated acute appendicitis on adults are still scarce and to our knowledge there is no reported data on the quality of life after antibiotic treatment for uncomplicated acute appendicitis. Lundholm et al. [44] reported long-term follow-up results with most of the recurrences occurring during the first year of follow-up. However, their report included patients from an earlier RCT, in which roughly half of the patients initially allocated to the antibiotic group crossed over to the surgery group, as well as 271 non-randomised patients with initial favourable antibiotic response to acute appendicitis, diagnosed without a standardised imaging protocol and without differentiating between uncomplicated and complicated appendicitis. Also, one study on children with an average follow-up of 4.3 years reported a median time for recurrence of 6 months [45]. The median time for recurrence, i.e. appendectomy, was 102 days in our APPAC trial [10] and based on the available information, most of the recurrences seem to occur within the first year, and it is therefore reasonable to use one-year follow-up for the primary endpoint evaluation. The late recurrence rates at 3, 5 and 10 years, which are defined as secondary outcome parameters, are inarguably essential in determining the optimal treatment for patients with uncomplicated acute appendicitis.

### Diagnosis of acute appendicitis

CT confirmed uncomplicated acute appendicitis is the inclusion criterion in this study. For that reason all patients evaluated for enrolment due to suspected acute appendicitis will undergo optimised CT imaging resulting in good diagnostic accuracy and minimised selection bias. The diagnostic criteria used in the CT imaging in the APPAC trial [10] with minor adjustments were chosen to be used as a base on the structured report of the APPAC II trial aiming for most accurate and reproducible differential diagnostics. When selecting patients for possible non-operative treatment, the accurate differential diagnosis between uncomplicated and complicated forms of acute appendicitis is of vital clinical importance and diagnostic contrast enhanced imaging with a high sensitivity in detecting patients with complicated acute appendicitis is needed. A recent radiological meta-analysis [22] stated specific CT imaging features most informative for complicated appendicitis and all these CT findings are used also as criteria for complicated appendicitis in the current APPAC II study. The used CT protocols (low-dose or standard) are thoroughly recorded and the radiologists use a standardised CT scan report sheet (Table 1) in order to facilitate standardisation of the diagnostic imaging, which has been shown to result in high reproducibility of objective CT findings achieving high diagnostic accuracy in an at-risk population [46]. This standardised recording enables thorough assessment of the accuracy of diagnostic CT imaging (low-dose and/vs. standard) used in this study, as well as the retrospective analysis of the accuracy of the existing scoring systems with clinical and laboratory findings used for diagnosing acute appendicitis.

In addition to the undisputed advantages in differential diagnostics between uncomplicated and complicated acute appendicitis, routine CT imaging in the diagnosis of acute appendicitis is also known to reduce the rate of negative appendectomies [47], surgical complications and treatment costs [33]. However, it cannot be disregarded that CT imaging exposes patients to harmful ionising radiation potentially increasing future cancer risk [34], which especially in the case of acute appendicitis requires attention as the majority of the patients are young adults. This is however an unavoidable downside in evaluating patients to possible non-operative treatment as the accurate differential diagnosis between uncomplicated and complicated acute appendicitis requires imaging [21, 28, 29]. There is accumulating evidence showing that low-dose CT protocols are as accurate as the standard protocols in diagnosing acute appendicitis [36, 48], thereby offering means to reduce the amount of ionising radiation resulting from the diagnostic CT imaging of acute appendicitis. Reducing the radiation dosage by using low-dose CT scan instead of a standard CT scan without compromising diagnostic accuracy warrants further active research. We decided to use standard 120 kV protocol for obese patients in the APPAC II trial if the automated voltage selection technique is not available due to our previous results on our OPTICAP phantom trial [38]. Also, Poletti et al. [49] stated that diagnostic accuracy of low-dose abdominal CT protocols may not yet be adequate in patients with BMI exceeding 30 kg/m^2^.

### Recurrent appendicitis after antibiotic therapy

All patients with clinically clear suspicion of appendicitis recurrence undergo laparoscopic appendectomy without further imaging. Patients with atypical or vague symptoms undergo regular clinical evaluation including possible imaging based on the assessment of the surgeon on call. This approach of direct appendectomy was chosen as the optimal primary treatment for uncomplicated acute appendicitis is still under active research and this approach enables the histopathological and microbiological assessment of removed appendix in order to further evaluate recurrent appendicitis cases after antibiotic treatment.

### Choice of the antibiotic regimen

The most common organism isolated from acute appendicitis is *Escherichia coli*, followed by *Klebsiella pneumoniae*, *Streptococci*, *Enterococci* and *Pseudomonas aeruginosa* [50]. Using the same antibiotic combination (i.v. ertapenem followed by p.o. levofloxacin and metronidazole) already tested effective and safe in the APPAC trial [10] was evaluated feasible for the standard antibiotic therapy, but the treatment duration was shortened from 3 days of i.v. + 7 days of p.o. to 2 and 5 days, respectively. The duration of the antibiotic treatment is seven days even though recent data shows that after adequate source control outcomes after short duration antibiotics for intra-abdominal infections are similar to those after a longer course of antibiotics [51], but as in the antibiotic treatment of uncomplicated acute appendicitis there is no source control, the duration of the antibiotic therapy was set at one week. As there is no oral ertapenem available, direct comparison between i.v. and p.o. antibiotics is not possible and thus the extent of the observed effects due to the antibiotic regimen used or the administrative route for the antibiotic in question cannot be reliably differentiated. However, the aim of the study was to evaluate a broad-spectrum antibiotic without the need for i.v. administration, which could be potentially feasible also for outpatient treatment in the future. We found moxifloxacin a practical choice for the p.o. monotherapy, as it is included in current practice guidelines for community acquired intra-abdominal infections [52], and has proven efficacy in intra-abdominal infections against facultative and aerobic gram-negative organisms as well as anaerobic bacteria [53, 54]. The once-daily administration of moxifloxacin was also regarded as an advantage. Further bacteriological analyses on the appendiceal microbial growth isolated from the study cases non-responsive to antibiotic treatment are needed in order to decide the best antibiotic regime in the future. Using broad spectrum intravenously administered antibiotics such as ertapenem increases the risk of developing antibiotic resistance also resulting in an increased need of hospital resources. However, treatment targeted only to patients with imaging-confirmed uncomplicated acute appendicitis and a limited treatment duration of 7 days is unlikely to be of great relative importance in the promotion of community bacterial resistance.

As we have some evidence suggesting spontaneous resolution of uncomplicated acute appendicitis [3, 16, 17], future research will determine the eventual need for antibiotics altogether. To date, however, the evidence on spontaneous resolution of appendicitis is so scarce that we will only have to recognise this view as a possible source of bias in our results and a double-blinded, placebo-controlled RCT is needed to more decisively differentiate the role of antibiotic treatment vs. spontaneous resolution of uncomplicated acute appendicitis.

In conclusion, this paper describes the design of a prospective multicenter randomised study that will evaluate the p.o. antibiotic monotherapy compared with i.v. + p.o. antibiotic therapy in terms of treatment efficacy, post-intervention complications, length of hospital stay and treatment costs. The inclusion of the patients has been initiated in April 2017 and the estimated completion of the enrollment will be in the fall 2018. APPAC II trial results will give further clinical knowledge on the debated role of antibiotics as the first-line treatment for a CT-confirmed uncomplicated acute appendicitis as well as assessment of possible optimisation of the antibiotic therapy for uncomplicated acute appendicitis.
